# Influence of Grain- and Grass-Finishing Systems on Carcass Characteristics, Meat Quality, Nutritional Composition, and Consumer Sensory Attributes of Bison

**DOI:** 10.3390/foods10051060

**Published:** 2021-05-12

**Authors:** Jessica Janssen, Kristi Cammack, Jerrad Legako, Ryan Cox, J. Kyle Grubbs, Keith Underwood, John Hansen, Carter Kruse, Amanda Blair

**Affiliations:** 1 Department of Animal Science, South Dakota State University, Brookings, SD 57007, USA; jessicakay401@gmail.com (J.J.); kristi.cammack@sdstate.edu (K.C.); judson.grubbs@sdstate.edu (J.K.G.); keith.underwood@sdstate.edu (K.U.); 2 Department of Animal and Food Sciences, Texas Tech University, Lubbock, TX 79409, USA; jerrad.legako@ttu.edu; 3 Department of Animal Science, University of Minnesota, St. Paul, MN 55108, USA; ryancox@umn.edu; 4 Turner Enterprises Inc., Bozeman, MT 59718, USA; John.hansen@tedturner.com (J.H.); Carter.kruse@retranches.com (C.K.)

**Keywords:** bison, carcass, finishing system, grain, grass, meat quality, nutritional composition, sensory, tenderness

## Abstract

The objective of this study was to determine the influence of two finishing systems (grain- or grass-finishing) on carcass characteristics, meat quality, nutritional composition, and sensory attributes of bison. Bison heifers were assigned to either a grain- or grass-finishing treatment for 130 days prior to slaughter. Carcass measurements, lean color and fat color were recorded. Striploins (*M. longissimus lumborum*) were collected for analysis of pH, fatty acid profile, cholesterol, proximate analysis, Warner-Bratzler shear force, cook loss, and consumer sensory evaluation. Grain-finished bison heifers had greater (*p* < 0.01) hot carcass weights, dressing percentage, ribeye area, backfat, and marbling scores compared to grass-finished heifers. Instrumental color values (L*, a*, b*) of the ribeye and a* value of backfat opposite the ribeye were greater (*p* < 0.01) for grain-finished heifers. Steaks from grain-finished heifers had increased (*p <* 0.05) crude protein and fat content and decreased (*p <* 0.01) moisture compared to grass-finished heifers. The grain-finishing system produced steaks with increased (*p* < 0.01) cholesterol and total fatty acids (mg/g of wet tissue). The grain-finished system produced more tender (*p* < 0.05) steaks, but consumer sensory ratings did not differ (*p* > 0.10) between treatments. These data indicate that finishing systems influence bison carcass characteristics, nutritional composition, and meat quality, but do not translate to differences in consumer preferences.

## 1. Introduction

Production and consumption of bison (*Bison bison*) has increased significantly since they were hunted to near extinction in North America during the late 1800s [1,2]. Currently it is estimated that there are approximately 400,000 bison in North America, including private, state, federal, and tribal herds [3]. Despite growing popularity, meat quality and consumer preferences for bison are not well characterized, which limits opportunities to expands markets. In addition, both grain- and grass-finishing systems are utilized within the industry, contributing to product variation.

Research describing the influence of finishing system on bison carcass traits and meat quality are lacking. Results from beef studies have generally shown that forage finishing results in leaner carcasses compared with grain finishing when cattle are harvested at similar ages [4,5,6]. Several beef studies have also reported that finishing system can impact meat quality [7,8,9,10,11,12], since the nutrient composition of the feed and amount of dietary energy available to the animal can modify carcass composition [13], including the amount of intramuscular fat and the fatty acid profile. Changes in intramuscular fat and fatty acid profile are known to influence the eating quality and flavor of beef [14,15,16,17,18]. Grain-finished beef is considered to have more acceptable flavor than forage-finished beef [16,19,20,21]. Changes in fatty acid profile can also impact nutritional quality. Food products containing increased ratios (greater than 0.45) of polyunsaturated fatty acids (PUFA) to saturated fatty acids (SFA) and lower ratios (less than 4.0) of n-6 to n-3 fatty acids may reduce the incidence of coronary artery disease [22]. Forage-finished beef has been reported to have an increased PUFA to SFA ratio and an improved n-6 to n-3 ratio [23,24].

Currently there is very limited research on the carcass characteristics produced across the bison industry, or the effects of common finishing systems on product outcomes. The objective of this study was to characterize the influence of finishing system (grain-finished or grass-finished) on carcass characteristics, meat quality, nutritional composition, and consumer preference for bison meat.

## 2. Materials and Methods

### 2.1. Animals, Carcass Evaluation, and Striploin Collection

Prior to treatment allocation, bison heifers (*Bison bison*; *n* = 208) were allowed to graze native range near central South Dakota [common vegetation included: western wheatgrass (*Pascopyrum smithii*), blue grama (*Bouteloua gracilis*), needle-and-thread (*Hesperostipa comata*), and green needlegrass (*Nassella viridula*)]. When heifers were approximately 24 months of age, they were randomly assigned to one of two finishing treatments: Grain- (*n* = 108) or grass- (*n* = 93). Grain-finished heifers were placed in an open lot (9290 m^2^; allowing approximately 93 m^2^ per animal) and provided ad libitum access to prairie grass and alfalfa hay bales placed in hay rings, as well as a concentrate mixture (83% corn, 17% dried distillers grain) placed in feed bunks for 130 days prior to slaughter. Grass-finished heifers were allowed to continue grazing native range pasture until harvest. Both finishing treatments had access to a custom loose mineral and vitamin supplement [Custom Mineral Mix: Product numbers: 602713 and 603652 (included Rabon for fly control May-October, 2018) Furst-McNess, Freeport, IL, USA].

At approximately 28 months of age, all heifers were transported (~720 km) to a commercial harvest facility and harvested over a two-day period. On the first day of slaughter, 47 head of grass-finished heifers and 54 head of grain-finished were slaughtered. On the second day of slaughter, 46 head of grass-finished and 54 head of grain-finished were slaughtered. After an approximately 20 h chilling period, carcasses were ribbed between the 12th and 13th rib and ribeye area, backfat thickness, marbling score, skeletal maturity, lean maturity, and external fat color were determined by United States Department of Agriculture (USDA) graders. Skeletal maturity was subjectively scored based on the ossification percentage of the thoracic cartilage buttons, and assigned a number (11, 7, 5, or −5) that corresponded with ossification percentages as follows: 0–24% (slight, 11), 25–49% (moderate, 7), 50–99% (hardbone, 5), or 100–200 (extreme hardbone, −5). Lean maturity was subjectively scored based on the lean color of the exposed ribeye, and assigned a number (11, 7, 5, 3, 1, or 0) corresponding to a color description as follows: bright red (11), moderately bright red (7), slightly bright red (5), red (3), pale red (1), or dark cutter (0). Fat color was subjectively scored based on the external fat color, and assigned a number (11, 7, 5, 3, or 1) that corresponded to fat color as follows: white (11), moderately white (7), slightly white (5), moderately yellow (3), and yellow (1). Additionally, objective color (L*, a*, b*) of the exposed ribeye area and the subcutaneous fat of the carcass surface opposite the ribeye were recorded using a handheld Minolta colorimeter (Model CR-310, Minolta Corp., Ramsey, NJ, USA; 50 mm diameter measuring space; D_65_ illuminant). A subsample (*n* = 60; 30 carcasses closest to the average hot carcass weight (HCW) for each harvest date per treatment) was selected and transported to a commercial processing facility. Striploins (*M. longissimus lumborum*) were removed from one side of each carcass, vacuum packaged, and transported in a refrigerated trailer (maintained at approximately 4 °C) back to the South Dakota State University (SDSU) Meat Laboratory.

### 2.2. Striploin Fabrication and pH

Striploin samples arrived at the SDSU Meat Laboratory at 2- or 3-days postmortem. Upon arrival, all striploins were removed from vacuum packages and trimmed of external fat. Ultimate pH was recorded at the posterior end of the striploin using a hand-held pH meter (Thermo-Scientific Orion Star, Beverly, MA, USA, Model# A221 and Star A321 Portable pH Probe). An approximately 1.27-cm slice was removed from the anterior end of each striploin to create an even surface. The remaining striploin was fabricated into 2.54-cm steaks, all of which were individually vacuum packaged and assigned for analysis. One steak was designated for proximate analysis, analysis of cholesterol content and fatty acid profile and was frozen immediately. Five steaks were designated for Warner-Bratzler shear force (WBSF). One steak was stored for 14 days at 4 °C and sheared without freezing (fresh). Four additional steaks were assigned to a 4-, 7-, 14-, or 21-day aging period, then frozen for approximately three months at −10 °C prior to shear force analysis. The 14-day aged fresh and frozen samples were utilized to compare the influence of freezing on bison steak tenderness. Two steaks designated for a consumer sensory panel were aged for 14 days and frozen.

### 2.3. Proximate Analysis

To determine proximate nutrient composition of the *M. longissimus lumborum* steaks collected from the anterior end of each striploin (at the junction between the 14th thoracic vertebrae and the 1st lumbar vertebrae) were thawed slightly and trimmed of excess external fat and accessory muscles, minced with a knife, submerged in liquid nitrogen, and powdered using a stainless-steel blender (Waring Products Division, Model# 51BL32, Lancaster, PA, USA). Homogenized samples were stored at −20 °C in plastic bags (Whirlpack, Nasco, Fort Atkinson, WI, USA) until chemical composition analyses. Percent crude fat and moisture were determine using ether extraction as described by Mohrhauser et al. [25]. Powdered samples were weighed (~5 g) into dried aluminum tins (FisherBrand, Pittsburgh, PA, USA, Cat.# 08-732-101), covered with dried filter papers (Whatman, Buckinghamshire, UK, Cat.# 1001-1055) and dried in an oven (Precision Scientific, Winchester, VA, USA, Cat.# 51220159) at 101 °C for 24 h. Dried samples were then placed into a desiccator (Scienceware, Wayne, NJ, USA, Cat.# 420320000) and samples were reweighed after cooling for at least 1 h. Proximate moisture content was calculated as the difference between pre- and post- drying sample weights and expressed as percent of the pre-drying sample weight. Dried samples were then extracted with petroleum ether in a side-arm Soxhlet extractor (ThermoFisher Scientific, Rockville, MD, USA) for a 60-h reflux period followed by evaporation under the laboratory hood at room temperature for 4 h and subsequent drying in an oven at 101 °C for 4 h. Dried, extracted samples were placed in desiccators to cool for 1 h and then reweighed. Proximate intramuscular fat content was calculated as the difference between pre- and post-extraction sample weight and expressed as a percent of the pre-extraction sample weight.

To determine ash percentage of each sample, duplicate powdered samples were weighed (approximately 3 g) into dried ceramic crucibles (COORSTEK, Golden, CO, USA, Cat. #60109) and placed into an oven at 101 °C for 24 h. Dried samples were then placed into a glass desiccator and samples were reweighed after cooling for at least 1 h, and then placed into a muffle furnace (Fisher Scientific Co., Pittsburgh, PA, USA, Model Series# 10-650) at 500 °C and ashed for 24 h. Ashed samples were removed and placed into a desiccator once the furnace cooled down to approximately 150 °C. Ashed samples were cooled in the desiccator for at least 1 h then reweighed. Proximate ash content was calculated as the difference between pre- and post-ashed sample weights and expressed as percent of the pre-ashed sample weight.

To determine protein content, duplicate powdered samples were weighed (approximately 250 mg) into crucibles and were subjected to dumas combustion by a nitrogen analyzer (Rapid Max N Exceed, Elementar, Hanau, Germany, Serial# 29161032). Percent protein content was determined based on the protein factor (6.25) multiplied by the percent nitrogen detected for each sample.

### 2.4. Cholesterol Determination

To determine cholesterol content of the *M. longissimus lumborum* muscle steaks collected from the anterior end of each striploin (at the junction between the 14th thoracic vertebrae and the 1st lumbar vertebrae) were thawed slightly and trimmed of excess external fat and accessory muscles, minced, submerged in liquid nitrogen, and powdered using a stainless-steel blender (Waring Products Division, Model# 51BL32). Homogenized samples were held at −80 °C in plastic bags (Whirlpack, Nasco) until used for cholesterol determination. The AOAC Official Method 994.10, Cholesterol in Foods, Direct Saponification-Gas Chromatographic Method [26] was used with modifications described by Dinh et al. [27]. Cholesterol standards were prepared at concentrations of 0.0125, 0.025, 0.05, and 0.1 mg/mL to construct a standard curve for cholesterol determination. An internal standard, 5α-cholestane (ACROS Organics, NJ, USA, Cat.# AC165602500), was used as a correction factor to standardize injection errors. All standards were diluted in high-grade toluene (ACROS Organics, Lot# B052366, UN1294), and were subjected to gas chromatographic system (GC) analysis before and after sequential sample analysis to obtain an average curve. Frozen steak samples were accurately weighed to 1.000 (to the nearest 0.001 g) and placed into 125-mL flat-bottom boiling flasks, followed by the addition of 2 mL of 50% potassium hydroxide (KOH) in water and 10 mL of 95% ethanol. Flasks were placed onto heated magnetic stir plates and the mixtures were boiled, stirred, and refluxed for at least 25 min, or until mixture was clear. Flasks were removed from the stir plates and allowed to cool to room temperature (~25 °C). Mixed solutions were transferred from the boiling flasks to separatory funnels, followed by the addition of 10 mL high-grade toluene and 1.0 N aqueous KOH. Funnels were shaken vigorously for at least 10 s. Mixtures were allowed to stand until the toluene layer was distinctly separated from the bottom aqueous layer. The bottom aqueous layer was discarded, and 5 mL of 0.5 N aqueous KOH was added, gently mixed, and allowed to stand until a clear separation of layers occurred. The bottom aqueous layer was again discarded. The remaining toluene layer was purified by four washes of 5 mL of deionized water. After each wash of deionized water, the solution was mixed, and allowed to stand for complete separation of layers, which allowed the bottom aqueous layer to be discarded before the next wash. The final toluene layer, which could be cloudy, was poured into a 50 mL test tube containing approximately 3 g of anhydrous sodium sulfate. Test tubes were shaken for approximately 5 s to remove excess moisture associated with the toluene. The mixture was allowed to stand until a visibly clear toluene solution appeared, with the anhydrous remaining as a white gelatinous bottom layer. Additional anhydrous was added if the final toluene layer remained cloudy after shaking and allowed to settle. The final purified extract was stored in test tubes with Teflon-lined caps under refrigeration. Prior to mixing, all solutions were brought to room temperature. In a 2.0 mL GC vial (Agilent Technologies, Santa Clara, CA, USA, Part No., 5188–6592, Batch No., GTG023112229), 0.5 mL of the clear toluene solution containing the extracted cholesterol was mixed with 0.5 mL of internal standard and subjected to GC analysis. 

Liberated cholesterol was quantified using the Agilent 6890N GC system and the DB-17 capillary column (30 m × 0.250 mm × 0.15 μm, Agilent Technologies Inc.). The DB-17 has mid-polarity and is suitable for analysis of free steroids. One microliter (1.0 µL) of analyte cholesterol mixture was injected into the GC system with split/splitless injector and flame ionization detector. The inlet temperature was 250 °C and split ratio was 50:1. The carrier gas was helium at 1.4 mL per min constant flow. The oven was programmed isothermally at 260 °C and held for 13 min. Total time for GC determination was 15 min. The detector was set at 350 °C with 450 mL per min airflow, 40 mL per min hydrogen flow, and 40 mL per min constant column and helium makeup flow. 

### 2.5. Fatty Acid Composition Analysis

For fatty acid methyl ester (FAME) analyses of the *longissimus dorsi* muscle samples were thawed slightly and trimmed of excess external fat and accessory muscles, minced, submerged in liquid nitrogen, and powdered using a stainless-steel blender (Waring Products Division, Model# 51BL32). Homogenized samples were held at approximately −80 °C in plastic bags (Whirlpack, Nasco) until FAME analyses. Frozen samples were accurately weighed to 1.000 (to the nearest 0.001 g) and processed to generate FAME according to procedures outlined by O’Fallon et al. [28]. Analysis of FAME was conducted by GC using an HP-88 capillary column (30m × 0.25 mm × 0.20 µm; Agilent Technologies, Palo Alto, CA, USA) and a flame ionization detector (FID). One microliter of sample was injected with a split ratio of 50:1. The oven method was as follows: 120 °C held for 1 min, increased to a temperature of 170 °C at the rate of 15 °C per min, held for 2 min, then increased to a temperature of 200 °C at the rate of 3 °C per min, held for 1 min, and finally increased to a temperature of 235 °C at a rate of 20 °C per min and held for 1 min. Hydrogen was used as the carrier gas. The FID was operated at 300 °C. Fatty acid methyl esters were identified and quantified by use of authentic standards (Supelco 37 Component FAME mix, Sigma-Aldrich, St. Louis, MO, USA). Concentrations of fatty acids were calculated and expressed on both a raw wet-weight, and percentage of total fatty acid basis.

### 2.6. Warner-Bratzler Shear Force and Cook Loss

Warner-Bratzler Shear Force was utilized to compare the tenderness of grass- and grain-finished bison, the influence of postmortem aging on tenderness of striploin steaks from grain- and grass-finished bison, and the influence of storage conditions (fresh versus frozen) on tenderness of bison striploin steaks. In preparation for WBSF, frozen steaks were thawed at 4 °C for 24 h before cooking. All steaks were weighed prior to cooking to an internal temperature of 71 °C. Steaks were cooked on an electric clamshell grill (George Forman 9 Serving Classic Plate Grill, Model GR2144P, Middleton, WI, USA). Internal temperature was monitored using a digital thermometer (Cooper-Atkins, Middlefield, CT, Model# 41-983430-5) placed near the geometric center of each steak. After cooking, all steaks were allowed to cool to room temperature before they were reweighed to determine cook loss. Cook loss was reported as a percentage of the raw weight using the following equation: [(raw weight − cooked weight)/raw weight] × 100. Cooked steaks were cooled at 4 °C for 24 h before removing 5 to 6 cores (1.27 cm in diameter) parallel to the muscle fiber orientation. Each core was sheared once perpendicular to the muscle fiber orientation and peak force was recorded [29]. A texture analyzer (Shimadzu Scientific Instruments Inc., Lenexa, KS, USA, Model EZ-SX) with a Warner-Bratzler attachment was used to determine peak force required to shear each core. An average shear peak force value was then reported for each steak.

### 2.7. Consumer Preference

A consumer sensory panel was conducted at the University of Minnesota Sensory Laboratory to determine subjective meat quality characteristics of grain- and grass-finished bison striploin steaks. A sub-sample (*n* = 46; steaks from the 23 carcasses closest to the average marbling score for each treatment) was utilized for the sensory panel. Random participants (*n* = 113) were recruited from the student and staff population of the University of Minnesota and included anyone who expressed an interest in participating in sensory tests. Participants were 18 years or older, had no food allergies or sensitivities, were willing to consume bison meat, and must have consumed any type of meat at least once a year. Participants were compensated for their time. The University of Minnesota’s Institutional Review Board (IRB) approved all recruiting and experimental procedures (IRB #6792). Sample steaks, aged 14 days and kept in frozen storage conditions approximately 10 months prior to analysis, were wrapped in aluminum foil, and allowed to thaw for 48 h before they were placed in an electric oven set to 204 °C. Internal temperature was monitored using a digital thermometer (Cooper-Atkins, Model# DTT361-01) placed near the geometric center of each steak. Steaks were cooked until they reached an internal temperature of 71 °C. Cooked steaks were allowed an approximate 3 min rest time before they were trimmed of external fat, placed into a grid cutter, and cut into 1-cm × 1-cm × 2.5-cm sample cubes. Cubes were held in porcelain double boilers, lined with aluminum foil, and heated to approximately 60 °C to maintain temperature before allocation to individual sample cups. Samples were transferred to lidded, 4 oz. foam cups with random 3-digit codes specific to each treatment code. The foam cups were held until served inside a proofing cabinet (Win-Holt NSF ETL, Syosset, NY, USA, Model #NHPL-1836C) set to a temperature of 54–60 °C and a humidity setting of 9. Each participant received two samples per treatment (samples from each treatment were presented to panelists simultaneously) and were provided with distilled water.

Participants were first asked to assess aroma liking. They were instructed to evaluate sample aroma by partially opening the sample lid and observing the aroma of the sample. Participants were then instructed to taste one of the sample cubes and rate it for overall liking, liking of flavor, and liking of texture. Participants were then instructed to taste the second piece and rate tenderness, juiciness, and off-flavor intensity. Liking ratings were made on 120-point labeled affective magnitude scales, with the left most end labeled ‘greatest imaginable disliking’ and the right most end labeled ‘greatest imaginable liking’. Intensity ratings were made on 20-point line scales with the left most ends labeled ‘none’ and the right most ends labeled ‘extremely intense’ for off-flavor, ‘extremely juicy’ for juiciness, and ‘extremely tough’ for toughness. Participants who rated the off-flavor at an intensity of 10 or more were required to answer the following open-ended question: “Please describe, as specifically as you can, what this off-flavor was”.

### 2.8. Statistical Analysis

Live body weight, dressing percent, carcass measurements, shear force, cook loss, storage conditions (fresh vs. frozen for cook loss and shear force analyses), fatty acid profile, cholesterol content, and proximate analysis data were analyzed using the MIXED procedures of SAS (SAS Inst. Inc., Cary, NC, USA). Subjective carcass measurements, including fat color, lean, and skeletal maturity, and all USDA Yield Grade data were analyzed using the GLIMMEX procedures of SAS for the main effect of finishing treatment. Kill date was included as a random effect, and peak steak cooking temperature was included as a covariate for shear force and cook loss. The interaction of storage conditions with finishing treatment was initially included but was not significant for shear force or cook loss and therefore omitted from the final model. Cook loss and shear force samples were subjected to different postmortem aging periods before frozen and were analyzed as repeated measures using the ante-dependence covariance structure in the MIXED procedure of SAS for effects of finishing treatment, aging, and their interaction; peak temperature was included as a covariate. The interaction of postmortem aging period with finishing treatment was not significant for shear force and therefore omitted from the model. Consumer preference data was analyzed using the MIXED procedures of SAS for the main effects of finishing treatment and serving order; time and panelist were used as random effects. For all attributes except toughness and juiciness ratings, serving order was not significant and omitted from the final model. Separation of least-squares main effect means was performed using LSD with a Tukey’s adjustment and assuming an alpha level of 0.05. Carcass served as the experiment unit for all carcass and meat quality analyses, and the individual panelists served as the experimental unit for sensory analysis.

## 3. Results

### 3.1. Carcass Characteristics

In the United States, bison are classified as a non-amenable or “exotic” species, carcass inspection is voluntary, and no established carcass yield or quality grading system exist for the specie. Because of these gaps in the U.S. bison industry, carcass measurements evaluated in this study were measured using standards utilized in determining yield and quality grades of beef carcasses. Additionally, the Canadian bison carcass grading system will be referenced when relevant. Research investigating bison carcass characteristics is very limited, and thus results from beef studies will be discussed to provide context. It is important to note that the anatomy and conformation of bison carcasses differ somewhat from beef carcasses. Fat distribution of bison carcasses is described as less uniform than beef and a higher percentage of fat is distributed over the rib primal in bison carcasses compared with beef [30]. Bison generally have lighter finished weights and HCW, a smaller ribeye area, decreased marbling deposition, and achieve market readiness at a later chronological age than beef cattle [30]. The slaughter age of 28 months in the present study is within the range of 24 to 31 months reported in other bison studies [1,31,32,33,34].

Live weight and carcass data are reported in Table 1. The USDA Agriculture Marketing Service reports indicate that the average dressed HCW for bison heifers is 270 kg [35], which closely aligns with the HCW of the grain-finished treatment (281 kg) in the current study. Carcass weight of bison heifers (229 kg) reported by Lopez-Campos et al. [36] is similar to the grass-finished treatment in the present study (226 kg). Ribeye area of bison heifers was 64.58 cm^2^ and 57.48 cm^2^ for grain- and grass-finished respectively, similar to the ribeye area of 60.5 cm^2^ for bison steers reported by Hawley [31]. Koch et al. [30] reported bison averaged 2.21 cm of backfat thickness, which is similar to the backfat thickness of the grain finished heifers (2.16 cm) in the present study. In Canada, bison carcasses exhibiting greater than 1.2 cm of backfat are classified as over-finished, and the desirable backfat thickness range for the Canadian bison grading system is 0.2 to 1.2 cm [37]. Therefore, the backfat thickness of heifers in the grass-finished treatment (0.89 cm) would be more ideal than the grain-finished heifers if evaluated according to the Canadian system. Marbling scores of bison heifers in the current study were 389 and 244 for grain- and grass-finished respectively. These results are similar to scores reported by Lopez-Campos et al. [36] for bison heifers (368) and by Koch et al. [30] for bison bulls (319). Marbling scores ranging from 200–400 would classify bison carcasses as “practically devoid” to “slight” amounts of marbling using the USDA beef quality grading system, therefore qualifying the carcasses as either Standard or Select [38]. The Canadian bison grading system does not consider marbling scores [39].

Grain-finished bison heifers had heavier (*p* < 0.0001) live and HCW compared to grass finished heifers (Table 1). Grain-finished heifers also had increased dressing percentage, ribeye area, backfat, kidney pelvic heart fat (KPH) and marbling scores compared to grass-finished heifers. However, proportions of carcasses in each Yield Grade category (based on the United States Department of Agriculture beef yield grade equation) did not differ (*p* > 0.05) between treatments. Results of this study are similar to studies investigating the effects of finishing systems on beef cattle. Duckett et al. [40] reported forage finished steers had lighter final body weight, HCW, and decreased dressing percentage compared with concentrate finished steers that were harvested at a similar number of days on feed. Neel et al. [6] also reported forage-finishing resulted in lighter carcass weights compared to concentrate finishing when harvested at a similar time endpoint.

Similar to the bison results in the present study, Duckett et al. [40] reported concentrate finished beef steers had increased ribeye area, fat thickness at the 12th rib, KPH, and marbling scores compared to forage finished steers. Other beef research supports the conclusion that concentrate finishing results in increased weights and yield related carcass characteristics, as well as more marbling [4,6,41,42]. Marbling is considered an important meat quality characteristic for beef due its positive influence on tenderness, juiciness, and flavor. Therefore, the amount of marbling present in the ribeye is an important factor utilized to determine the quality grade of beef carcasses in the United States, and previous beef studies indicate that marbling content can be increased by feeding a higher concentrate diet [13,40,43]. However, the benefit of marbling to bison palatability is unknown.

### 3.2. Carcass Maturity and Subjective External Fat Color

There was no difference (*p* > 0.05) in the percentage of grain- and grass-finished bison heifers classified as ‘extreme hardbone’ (100–200% ossification) or ‘moderate’ (25–49% ossification) for skeletal maturity (Table 1). There was a tendency for a greater percentage (*p* = 0.0582) of grain-finished heifers to be classified as ‘hardbone’ (50–99% ossification) compared to grass-finished. A greater (*p* = 0.0031) percentage of grass-finished heifers were classified as ‘slight’ (0–24% ossification) for skeletal maturity compared to grain-finished. Overall, the greatest percentage of grass-finished heifers were classified as ‘slight’ (44.88%), while carcasses from grain-finished heifers were more distributed amongst ‘slight’ (24.32%), ‘moderate’ (36.84%), and ‘hardbone’ (28.69%) classifications. Regardless of finishing system, the ‘extreme hardbone’ category included the lowest percentage of bison heifers (7.71 and 6.25% for grain- and grass-finished respectively). Skeletal maturity has also been shown to increase in beef cattle as the percentage of concentrate in the diet is increased [44].

There was no difference (*p* > 0.05) in the percentage of grain- and grass- finished bison heifers classified as ‘red’, ‘slightly bright red’, or ‘moderately bright red’ for lean maturity (Table 1). An increased percentage (*p* = 0.0116) of grass-finished heifers were classified as ‘pale red’ compared to grain-finished heifers, while an increased percentage (*p* < 0.0001) of grain-finished heifers were classified as ‘bright red’ compared to grass-finished heifers. Overall, the greatest percentage of grain-finished heifers were classified as ‘bright red’ (41.64%), while carcasses from grass-finished heifers were more distributed amongst ‘red’ (24.73%), ‘slightly bright red’ (22.58%), and ‘moderately bright red’ (30.11%) classifications. Regardless of finishing system, the fewest carcasses were classified as ‘pale’ (0.74 and 9.97% for grain- and grass-finished respectively). The relationship between skeletal and lean maturity results indicate that grain-finished bison heifers exhibit an increased physiological maturity compared to grass-finished heifers at a similar chronological age.

There was no difference (*p* > 0.05) in the percentage of heifers classified as ‘slightly white’ for external fat color (Table 1). An increased percentage (*p* < 0.0001) of grass-finished heifers were classified as ‘moderately yellow’ compared to grain-finished heifers, while an increased percentage (*p* < 0.0001) of grain-finished heifers were classified as ‘moderately white’ compared to grass-finished heifers. Overall, the majority of grain-finished heifers were classified as ‘moderately white’ (64.89%), while majority of grass-finished heifers were classified as ‘moderately yellow’ (52.67%). Van Elswyk and McNeill [45] reviewed the impacts of forage versus grain finishing diets in beef and reported grass-fed beef to have increased yellowness of external fat. This is likely due to increased β-carotene deposition within adipose tissue of forage finished animals [5,40].

Due to their unique carcass characteristics, Canada has an established bison grading system [39] with 10 grades (A1–4, B1–3, and D1–3) dispersed into two different maturity classes (Maturity Class I, youthful; includes A1–4 and B1–3) and (Maturity Class II, mature; includes D1–3). Physiological maturity is determined by the degree of ossification present on the cartilage caps over the ends of the 9th, 10th, and 11th thoracic processes, where youthful carcasses have 80% or less ossification of the caps and mature carcasses have greater than 80% [37]. The Canadian grading system relates animal maturity, or age, directly to tenderness, in which ‘youthful’ carcasses are most tender. Utilizing the Canadian bison grades, a majority of carcasses in this present study would be classified as ‘youthful’; however, a greater percentage of grass-finished would fall into this classification than grain-finished heifers (74.78 versus 61.60%, respectively). A greater percentage of grain-finished bison heifers would be classified as ‘mature’ compared to grass-finished (36.40 versus 23.44%, respectively).

Other grade factors included in the Canadian grading system are degree of muscle color (lean maturity) and external fat color [39], which influence consumer acceptance and shelf-life. Therefore, bright red muscle color and white to amber fat color is preferred for carcasses in the A1–A4 and B1 grades, compared to a dark red muscle and yellow fat colors, which would be classified as B2 or B3 grades. When referencing the Canadian system, grain-finished carcasses in this study would be more desirable for fat and muscle color, as a majority were classified as moderately white for external fat color and bright or moderately bright red for lean muscle color compared to grass-finished.

### 3.3. Objective Color and Ultimate pH

Instrumental color values (L*, a*, b*) of the exposed ribeye and a* value of the external subcutaneous fat opposite the ribeye were increased (*p* < 0.0001; Table 2) for grain-finished heifers. However, L* and b* values of subcutaneous fat opposite the ribeye were increased (*p* < 0.0001; Table 2) for carcasses from the grass-finished system. Finishing system did not influence (*p* > 0.05; Table 2) ultimate pH of bison striploins.

In a comparison between bison and beef, Koch et al. [30] reported that bison muscles were darker than beef. The influence of finishing system on objective color of beef is generally in agreement with the current study reporting lighter lean color (greater L*) for beef finished on a concentrate diet as opposed to forage finished [4,40,41,42]. Duckett et al. [4] hypothesized that the darker lean color of forage finished beef was related to increased muscle pH; however, no differences were detected in pH in the current study. Others have attributed darker lean color to increased myoglobin content [9], possibly caused by increased physical activity of forage finished animals compared to animals finished in a feedlot [46]. In contrast to the present study, Duckett et al. [40] reported no difference in longissimus muscle a* or b* values between beef finishing systems. This could be due to differences in specie and diet composition between the two studies. Similar to this present study, Duckett et al. [40] reported that a* values of the subcutaneous backfat were increased for grain-finished beef, while the b* values were increased for forage-finished beef. However, in contrast to the present study no differences in L* values of the subcutaneous backfat of beef finished in different systems were reported [40].

Chail et al. [47] and French et al. [16] compared beef cattle finished on a forage diet in a grazing system to cattlse finished on a concentrate diet in a feedlot system and also reported no difference in ultimate muscle pH between treatments. In contrast, Duckett et al. [40] and Muir et al. [13] detected higher ultimate pH in grass-fed beef. French et al. [15] suggested that grass-fed steers were more susceptible to pre-slaughter stress than grain-finished, because the grain-finished steers were more accustomed to handling and penning. Bison heifers used in the present study were accustomed to various handling practices, received the same pre-slaughter handling, were the same age, and were killed within a two-day period, all of which may contribute to the lack of difference in pH.

### 3.4. Proximate Chemical Composition 

Steaks from grain-finished heifers had increased (*p* < 0.05) crude protein and fat content but decreased (*p* < 0.0001) moisture content compared to steaks from grass-finished bison heifers. Percentage of ash did not differ (*p* > 0.05) between finishing treatments (Table 3). 

These results closely follow compositional values for bison reported by Marchello and Driskell [1] and Marchello et al. [33]. Studies comparing the protein content of grass- and grain-finished bison are lacking. However, the protein content of grass-finished bison ribeye samples reported by Marchello and Driskell [1] was 21.5%, whereas the protein content of grain-finished bison ribeye samples reported by Marchello et al. [33] was 22.1% supporting the findings of this study that grain-finished bison have increased protein content compared to grass-finished. Results comparing grass- and grain-fed beef reported no difference in protein content between treatments [5,40,43]. The mechanism responsible for differences in protein content of bison between treatments is unknown. Overall, the limited studies on bison meat composition suggest that bison is lower in fat content (1.3–5.0%) than beef (3.0–10%) [1,30,31,33,48,49], which may be related to a greater percentage of bison that are grass-finished and the lack of genetic selection for marbling. Grain-fed animals generally consume high levels of energy in a high concentrate diet, which allows excess energy to be used to develop intramuscular fat [43]. Results comparing grass- and grain-fed beef also reported no difference in ash content between treatments, but a decrease in total fat content and subsequent increase in percent moisture of grass-finished compared to grain-finished samples [43]. This relationship between fat and moisture content has been reported by others investigating the proximate analysis of meat samples [7,50].

### 3.5. Cholesterol Content

The grain-finishing system produced steaks with increased (*p* = 0.0073) cholesterol content compared to grass-finished (Table 3). Cholesterol content was 54 and 51 mg/100 g for grain- and grass-finished heifers, respectively. These values are lower than the cholesterol values of bison bulls (66 and 65 mg/100 g for grain- and grass-finished, respectively) reported by Marchello and Driskell [1], but this is likely due to differences in sex or the fact that several cuts (ribeye, top sirloin, top round, and shoulder clod) were averaged in that study compared to only the striploin in the current study.

Cholesterol is a major component of animal plasma membranes because it is a vital structural component of cell membranes and the precursor of bile acids and steroid hormones [51]. Yet cholesterol is perceived to have negative effects on health, resulting in public concern over the cholesterol content in red meat products [52]. Eichhorn et al. [53] determined that adipose tissue contains about two times as much cholesterol as muscle tissue. However, all steaks in this study were trimmed of all external fat, and therefore the only fat source was from intramuscular fat. Intramuscular fat has been reported to contain less cholesterol than intermuscular fat [54]. It has been suggested that beef finished on grass yields steaks that are lower in cholesterol compared to those from a grain-finished system [55], but this is not consistent across studies. Some beef studies reported no difference in cholesterol between grass and grain treatments [5,40,43], however Rule et al. [34] reported reduced cholesterol content of grass-finished beef steaks from the round and chuck compared to grain-finished. Rule et al. [34] also reported cholesterol content was lower for the longissimus dorsi, semitendinosus, and supraspinatus muscles of range-raised bison compared to feedlot finished bison. When comparing the cholesterol content of muscles across different species (bison, elk, and beef) raised using different finishing systems, Rule et al. [34] reported that cholesterol content was lowest in the longissimus dorsi of range-raised bison compared to the other species and feedlot finished bison. However, the different dietary and species groups used by Rule et al. [34] included animals of various ages and sexes, which could also have impacted results. Ultimately, for meat to be classified as ‘lean’ according to the United States Dietary Guidelines, it must contain <95 mg/100 g cholesterol [56]. Therefore, bison steaks from both finishing systems in the present study would qualify as lean as they were well under the minimum requirement. 

### 3.6. Fatty Acid Profile

The majority of fatty acids evaluated were influenced (*p* < 0.05) by finishing treatment, with the exception of C12:0, C16:1 trans, C18:1 trans, C24:1n9, C18:2 trans, C18:3n3 (linolenic acid), C20:2, C20:6n3, C22:3, and C22:6n3 [docosohexanaenoic acid (DHA)] when reported on mg/g raw tissue basis (Table 4), and C10:0, C14:0, C17:0, and C24:1n9 when reported on a percentage of total fatty basis (Table 5). Grain-finished bison produced steaks with greater (*p* < 0.05) total concentrations of saturated fatty acids (SFA), monounsaturated fatty acids (MUFA), polyunsaturated fatty acids (PUFA), and overall total lipids (mg/g of wet tissue) compared to grass-finished. However, when expressed as a percentage of total lipid, grass-finished samples had increased total concentrations of PUFA (*p* < 0.0001) and SFA (*p* = 0.0219), while MUFA remained elevated (*p* < 0.0001) in grain- compared to grass-finished steaks. 

Results of this study are similar to studies investigating the effects of finishing systems on beef cattle. Beef studies reviewed by Van Elswyk and McNeil [45] revealed that SFA content, when reported as percent of total fatty acid basis, is increased in grass-fed and decreased in grain-fed. However, given that grass-fed beef is generally lower in total fat content, this percentage does not translate into an increased intake of total SFA in a g/100 g serving size, and thus grain-fed was reported to have increased SFA on a serving size basis. Rule et al. [34] compared the nutrient composition of bison placed on different finishing systems. When reported on a total fat percentage basis, grass-finished bison also had increased total SFA and PUFA content but decreased MUFA when compared to grain-finished bison [34].

Oleic acid (C18:1n9cis) is the predominate fatty acid in meat [57]; consequently, it was not surprising that oleic acid concentrations comprised the majority of both the grain- and grass-finished profile in the current study. Concentrations of oleic acid in bovine adipose tissue are dependent upon the activity of delta-9 desaturase, which is the enzyme responsible for the conversion of all SFAs to their respective MUFAs [58]. The decreased MUFA content of grass-finished beef is likely due to the effect of desaturase enzyme activities [58]. As intramuscular lipid accumulates, there is an associated elevation in the concentration of oleic acid, ranging from 30% to 50% of total adipose tissue fatty acids [59]. Results from the current study fall within this reported range; oleic acid concentrations were 45.60% for grain-finished and 37.38% for grass-finished bison steaks. Increased oleic acid concentrations for grain-finished bison steaks is supported by an increased presence of intramuscular fat content reported both by subjective and chemical evaluations. 

Forage feeding systems generally result in an increase in the PUFA to SFA ratio in ruminants [34,60]. However, overall content of PUFAs within red meat is generally low, only averaging only 5% in beef species [61]. However, results in the current study indicate bison PUFA concentrations well above 5% of total fatty acids regardless of finishing systems (13.75% and 20.53%, for grain- and grass-finished respectfully). Larick et al. [62] reported that bison had decreased total fat content but increased PUFAs compared to Bos taurus and Bos indicus cattle. Rule et al. [34] reported that range-fed bison, beef, and elk cows had similar fatty acid compositions, specifically the n-3 and n-6 PUFAs, and that range-fed beef and bison cows had greater proportions of PUFA compared to feedlot finished contemporaries.

Grain-finished bison steaks had an increased (*p* < 0.0001) n-6 to n-3 ratio but a decreased (*p* = 0.0006) PUFA to SFA ratio compared to grass-finished steaks. Diets with greater ratios of PUFA to SFA (greater than 0.45) and lower n-6 to n-3 ratios (less than 4.0) may reduce the incidence of coronary artery disease in consumers [22]. Both grain- and grass-finished bison steaks in this study had an n-6 to n-3 ratio greater than 4.0, yet grass-finished steaks had a significantly lower (*p* < 0.0001) ratio than grain-finished (4.64 to 5.74 for grass- and grass-finished, respectfully). Grass-finished steaks also had an increased (*p* = 0.0006) PUFA to SFA ratio compared to grain-finished (0.58 to 0.41 for grass- and grain-finished respectively). Grass finishing systems generally result in a decreased n-6 to n-3 ratio in ruminants [34,60]. Rule et al. [34] reported that samples from the longissimus dorsi of range fed bison had an n-6 to n-3 ratio of only 1.94, while the feedlot finished bison had a ratio of 5.73, which is similar to grain-finished steaks in the present study. However, the total proportions of PUFA reported by Rule et al. [34] (16.5% and 10.7% for range finished and feedlot finished bison, respectively) were less than the proportions reported in the current study (20.53% and 13.75% for grass- and grain-finished heifers respectively). As a result, the PUFA to SFA ratio reported by Rule et al. [34] (0.40 and 0.29 for range finished and feedlot finished bison respectively) was less than the ratio reported in this study. The differences between this study and ratios reported by Rule et al. [34] could be due to differences in animal age, sex, or diet composition.

### 3.7. Warner-Bratzler Shear Force 

**Figure 1:**
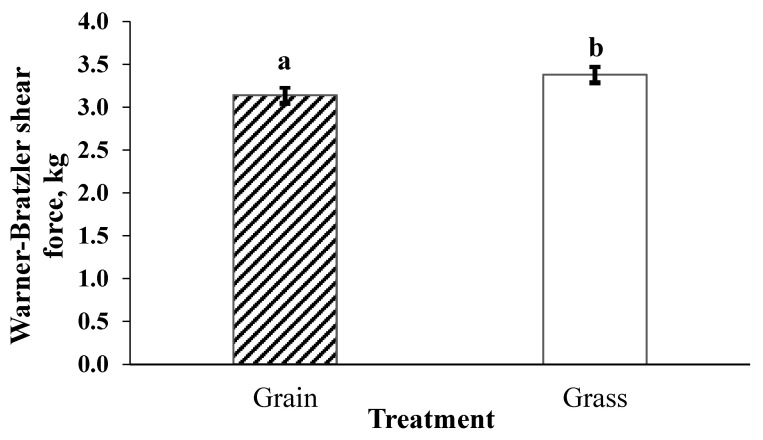
Least square means for the effect of finishing system on tenderness of bison striploin steaks. Treatments: GRAIN = bison heifers backgrounded on pasture and finished for 130 days with ad libitum access to grass hay, alfalfa, and a corn and dry distiller’s grain concentrate prior to slaughter. GRASS = bison heifers remained on pasture until slaughter. All steaks stored frozen prior to analysis. a,b means lacking a common superscript differ *p* < 0.05.

**Figure 2:**
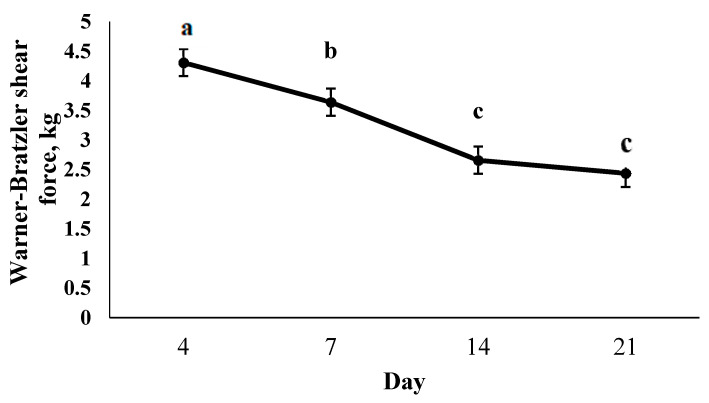
Least square means for the effect of postmortem aging on tenderness of bison striploin steaks^.^ Steaks were from grain- and grass-finished bison heifers and stored frozen prior to analysis. a,b,c means lacking a common superscript differ *p* < 0.05.

The grain-finished system produced more tender (*p* = 0.0131) steaks than grass-finished (Figure 1). Tenderness of all bison steaks improved (*p* < 0.0001) with postmortem aging (Figure 2). Steaks aged 4 days were toughest (*p* < 0.0001), followed by 7 days (*p* = 0.0246). Steaks aged 14 days were more tender than 4- and 7-day aged but did not differ (*p* > 0.05) from 21-day aged samples. 

It is well established that beef tenderness increases during postmortem storage of carcasses at refrigerated temperatures [63]. A factor involved in this increase in tenderness is postmortem loss of structural integrity of myofibrils [64,65] and other cytoskeletal elements [66] of the muscle cell. Tenderization occurs at a relatively rapid rate until 3 to 7 days postmortem, and then the rate diminishes with time, such that the improvement in tenderness of beef loins after 7 to 10 days is relatively small compared to the first 10 days [63,64,67,68]. Bison steaks in the current study appear to follow these postmortem aging trends, as tenderness improvements were observed until 14 days postmortem, then remained stable from 14 to 21 days postmortem.

Studies comparing grass-fed and grain-fed beef concluded that grass-fed beef was less tender than grain-finished [45,69], which was suggested to be partially due to decreased MUFA deposition resulting from the effects of delta-9 desaturase enzyme activity [58]. Delta-9 desaturase is responsible for the conversion of all SFAs to their respective MUFAs [58]. Early research demonstrated that MUFAs, specifically the concentration of oleic acid (18:1n-9), in beef is positively correlated with its overall palatability [70,71]. This improvement in palatability may be related to fat softness because beef lipids enriched with oleic acid have lower melting points [59,72,73]. In the present study, grain-finished bison produced steaks with increased concentrations of oleic acid both on a mg/g wet tissue basis and on a percentage of total fatty acids basis compared to grass-finished steers. Additionally, carcasses from grass-fed beef have been reported to have less subcutaneous fat, which increases the risk for cold shortening and reduces postmortem proteolysis, two factors that would reduce tenderness [69]. It is also proposed that animals finished on a sub-optimal plane of nutrition have an increased proportion of connective tissue associated with smaller muscle fibers, which exerts a negative influence on tenderness [69]. Grass-finished bison heifers in the present study did exhibit a smaller ribeye area compared with grain-finished heifers. Aberle et al. [74] and Fishell et al. [75] determined that pre-slaughter feeding and growth rate directly affect collagen stability and tenderness of beef. Cattle fed high energy diets experience rapid rates of protein synthesis, and therefore the meat produced from these animals would be expected to contain a large proportion of newly synthesized, heat-labile collagen [74,75]. Shimokomaki et al. [76] reported that changes in collagen crosslinking are related more closely to growth rate and animal maturity than chronological age. Hall and Hunt [77] proposed that cattle fed low energy diets grow at slower rates than cattle fed high energy diets. At a certain chronological age, forage-fed cattle would be physiologically less mature than their grain-fed contemporaries, and as a result, cattle quickly reaching maturity are likely to contain more soluble collagen and have more tender meat. In the present study all heifers were slaughtered at a common age (28 months), and a majority of grain-finished heifer carcasses were in the ‘moderate’ and ‘hardbone’ classifications for skeletal ossification, while more grass-finished carcasses were classified as ‘slight’. While the current study did not assess differences in collagen content, delta-9 desaturase activity, or proteolysis between samples from grain- versus grass-finished bison heifers, future studies could evaluate these factors to determine the mechanism by which tenderness is improved in grain-finished bison.

### 3.8. Cook Loss

**Figure 3:**
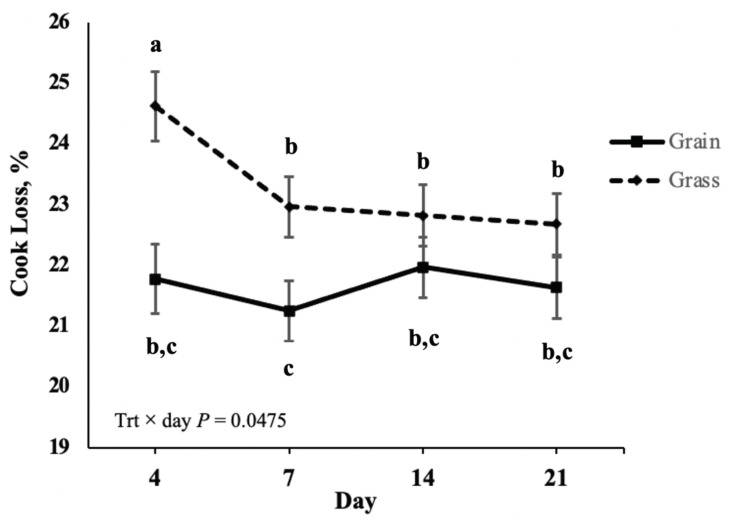
Least square means for the interaction of postmortem aging days and finishing system on cook loss of bison striploin steaks^.^ Treatments: GRAIN = bison heifers backgrounded on pasture and finished for 130 days with ad libitum access to grass hay, alfalfa, and a corn and dry distiller’s grain concentrate prior to slaughter. GRASS = bison heifers remained on pasture until slaughter. All steaks were stored frozen prior to analysis. a,b,c Means lacking a common superscript differ *p* < 0.05.

Cook loss was affected (*p* = 0.0475) by the interaction of finishing treatment with aging period (Figure 3). 

Overall, grain-finished steaks had less (*p* < 0.0001) cook loss than grass-finished steaks. Cook loss decreased for grass-finished from days 4 to 7 (*p* = 0.0468) but remained stable from days 7 to 21 (*p* > 0.05). Cook loss of grain-finished steaks did not differ between aging days (*p* > 0.05). All grain-finished steaks had decreased cook loss compared to 4-day grass-finished steaks; however, only 7-day grain-finished steaks had decreased cook loss compared to grass-finished steaks aged 7, 14, and 21 days. 

Bruce et al. [78] reported that beef longissimus thoracic steaks aged 14 days had increased cook loss compared to those aged for 1 day. Increased cook loss of aged steaks may be influenced by protein degradation during the aging process [79]. Additionally, as reported above, proximate analyses revealed that grass-finished steaks had increased moisture content, but decreased fat content compared to grain-finished steers. These differences in moisture and fat content between steaks could help explain cook loss differences between finishing treatments, as the moisture content is typically reduced in cuts with greater total fat content [80]. Additionally, increased intramuscular fat content lubricates the muscle fibers and fibrils, creating an insulation barrier during the application of high-temperature, dry-heat methods of cooking, and/or a greater degree of doneness without adversely affecting the palatability of the meat [81]. 

### 3.9. Influence of Storage Condition (Fresh vs Frozen) on Tenderness and Cook Loss

Bison steaks kept in frozen storage conditions had improved tenderness (*p* < 0.0001) but increased (*p* = 0.0001) cook loss compared to bison steaks kept in fresh storage conditions (Table 6). Shear force results are in agreement with Lopez-Campos et al. [36] who reported that shear force values of striploin steaks from bison bulls and heifers aged for 20 days and then frozen were decreased compared to steaks aged for 20 days and sheared fresh. Others have also concluded that frozen storage improves tenderness of beef [82,83] and lamb [84]. Shanks et al. [83] suggested that freezing results in intracellular ice formation, which causes a physical disruption of muscle cells, and Hiner et al. [85] suggested that freezing causes muscle fibers to rupture and induces stretching and rupture of connective tissues. It is possible that storage temperature and/or duration of frozen storage may affect the amount of intracellular ice formation and physical disruption occurring in muscle, and thus the extent to which freezing influences tenderness [83]. Smith et al. [86] reported freezing for a duration of 3 to 6 weeks had no effect on tenderness but reported that WBSF values decreased for beef stored frozen for 4 months.

Shanks et al. [83] reported no effect of storage conditions on cook loss of beef striploin steaks aged 14-, 21-, or 35-days postmortem and suggested that as meat ages and proteins degrade, muscle loses its inherit ability to hold moisture. However, cellular damage due to freezing may have outweighed this effect, and thus there would be little change in cook loss following freezing for steaks that were aged for longer period of time [83]. Despite results reported by Shanks et al. [83], others have reported that beef steaks held in frozen storage conditions have increased cook loss values [87,88,89]. In the United States, the average range for aging period for fresh beef at retail is 18–22 days, based on postmortem fabrication times reported in the 1991 and 1998 National Beef Tenderness Surveys [90,91]. The majority of beef tenderness research is conducted on steaks aged 14 to 21 days to simulate industry conditions [83]. Currently, there are no national surveys reporting average aging periods for fresh bison from fabrication to retail.

### 3.10. Consumer Preference

No treatment differences (*p* > 0.10) were detected by consumer panelists for overall liking, aroma liking, flavor liking, texture liking, toughness intensity, juiciness intensity, or off-flavor intensity of bison steaks (Table 7). The liking ratings were made on 120-point labeled affective magnitude scales ranging from greatest imaginable disliking to greatest imaginable liking. Consumer responses showed that all scores ranged from “like slightly” to “like moderately.” Intensity ratings were made on 20-point line scale with the left most ends labeled none and the right most ends labeled extremely juicy, extremely tough, and extremely intense for off-flavor. Means for intensity ratings were less than 10 for each attribute. Off-flavor intensity scores were the lowest, while juiciness scores were the greatest. Participants that rated off-flavor intensity at 10 or above were required to describe the off-flavor. An off-flavor intensity of greater than 10 was reported by 12.39% of participants (*n* = 14) for grain-finished and 10.61% (*n* = 12) for grass-finished. Common off-flavor descriptions for grass-finished steaks included: sour, rancid, liver, gamey, and fishy, while off-flavor descriptions for grain-finished steaks included: metallic, bitter, and sour.

Koch et al. [30] utilized a trained sensory panel and reported bison steaks to be more tender than beef, but no differences in objective shear force values were reported. The trained sensory panelists also indicated that bison meat had an intense off-flavor compared to beef, and the off-flavors described included ammonia, metallic, and gamey [30]. A similar trained sensory panel comparing shortloin steaks from bison to steaks from Bos taurus and Bos indicus cattle also suggested that bison samples exhibited more off-flavor and aftertaste presence compared to both cattle species [62]. These flavor notes were characterized as increased levels of ammonia, bitter, gamey, liverish, old, rotten, and sour [62]. Those flavor differences could be the result of fatty acid composition, specifically the increased PUFA content measured in bison compared to both cattle species. Polyunsaturated fatty acids can be responsible for the development of an oxidized flavor during storage [92], or warmed over-flavor in meats [93], and they are degraded during cooking [94]. Steaks from grass-finished bison heifers had increased PUFA concentrations, but no differences in off-flavor intensities were found.

Despite differences reported in shear force values, there were no differences (*p* = 0.2073) in consumer sensory response for toughness scores between bison finishing systems in the present study. It is important to note that as there was no aging day x treatment interaction for WBSF, and the WBSF values reported are main effect means including all aging periods (4, 7, 14, and 21 d). Steaks utilized for the sensory panel were aged for 14 days. The shear force values for the 14 day samples were 2.54 and 2.74 kg for grain- and grass-finished steaks, respectively. The American Society for Testing and Materials (ASTM) beef tenderness claim standards include a minimum tenderness threshold value (MTTV) of 4.4 kg for WBSF and is representative of instrumental and sensory research conducted for tender beef classification [95]. The shear force results in the current study, regardless of finishing system, are well below the MTTV. Further, a 0.5 kg difference in WBSF values represents the difference in shear force that the average consumer can detect when consuming meat [95]. Given the 14-day aged shear force values of this study, it is not surprising that the panelists were not able to detect tenderness differences between finishing systems. Additionally, Miller et al. [96] classified steaks with a shear force value < 3.0 kg to be very tender, which could allow for premium opportunities. Bison steaks from both finishing systems aged for at least 14 days were below 3.0 kg, indicating favorable eating quality characteristics.

## 4. Conclusions

Collectively these data indicate that finishing system impacts composition of bison carcasses, nutrient profile of bison meat, and measures of meat quality. Grain-finished bison heifers had increased carcass weights, backfat thickness, ribeye area, and marbling compared to grass-finished heifers. Finishing system influenced nutrient content and fatty acid composition, which may have consumer health implications, as grass-finished bison steaks exhibited a decreased cholesterol content, percent fat, and n6 to n3 fatty acid ratio when compared to grain-finished bison steaks. Steaks from grain-finished bison heifers were more tender and exhibited decreased cook loss compared to grass-finished. Differences exhibited in carcass and meat quality characteristics did not translate to differences in consumer preferences. Overall shear force and sensory results from this study indicate that bison produced from either grain- or grass-finishing systems can provide a favorable eating experience. Bison producers will need to recognize the variation in fat content, nutritional profile, and tenderness created by different production systems, and could utilize this information to differentiate products with specific attributes to more clearly market bison meat products from each finishing system.

## Figures and Tables

**Table 1:** Least squares means for effect of finishing system on live weight and carcass characteristics of grain- or grass-finished bison heifers.

Variable		GRAIN ^1^	GRASS ^1^	SEM ^2^	*p*-Value ^3^
Live weight, kg		445.93	378.40	2.962	<0.0001
Hot carcass weight, kg		281.43	226.42	2.285	<0.0001
Dressing percentage, %		63.09	59.81	0.234	<0.0001
Ribeye area, cm^2^		64.58	57.48	0.768	<0.0001
Backfat thickness, cm		2.16	0.89	0.084	<0.0001
Kidney, pelvic, and heart fat, %		2.56	0.87	0.069	<0.0001
Marbling score ^4^		389.35	243.67	9.924	<0.0001
Yield Grade ^5^					
	YG 2	5.56	55.91	5.148	0.0965
	YG 3	29.63	19.35	4.394	0.3435
	YG 4	46.30	3.23	4.798	0.1195
Skeletal maturity ^6^					
	Extreme Hardbone (100–200%)	7.71	6.25	6.140	0.6655
	Hardbone (50–99%)	28.69	17.19	4.771	0.0582
	Moderate (25–49%)	36.84	29.90	8.118	0.3033
	Slight (0–24%)	24.32	44.88	8.617	0.0031
Lean maturity ^6^					
	Pale Red	0.74	9.97	7.883	0.0116
	Red	5.56	24.73	4.474	0.1746
	Slightly Bright Red	19.44	22.58	4.336	0.6824
	Moderately Bright Red	32.41	30.11	4.757	0.7854
	Bright Red	41.64	7.49	6.377	<0.0001
Subjective external fat color ^7^					
	Moderately Yellow	1.84	52.67	6.593	<0.0001
	Slightly White	7.41	24.73	4.474	0.1918
	Moderately White	64.89	4.23	34.960	<0.0001

^1^ Treatments: GRAIN = bison heifers (*n* = 108) backgrounded on grain and finished for 130 days with ad libitum access to grass hay, alfalfa, and a corn and dry distiller’s grain concentrate prior to slaughter. GRASS = bison heifers (*n* = 93) remained on pasture until slaughter. ^2^ Standard error of the mean. ^3^ Probability of difference among least square means. ^4^ Marbling score: 100 = Practically Devoid^0^, 200 = Traces^0^, 300 = Slight^0^, 400 = Small^0^. ^5^ Yield Grade calculated according to USDA beef grading system; GLIMMIX analysis failed to converge for USDA Yield Grade 1 (*n* = 20) or 5 (*n* = 20). ^6^ Skeletal maturity and lean maturity assigned by USDA. GLIMMIX analysis failed to converge for Lean Maturity category ‘dark cutter’ (*n* = 3). ^7^ Subjective External Fat Color assigned by USDA. GLIMMIX analysis failed to converge for Yellow (*n* = 13) or White (*n* = 34) categories.

**Table 2:** Least squares means for effect of finishing system on objective color measurements and ultimate pH of grain- or grass-finished bison heifers.

Variable		GRAIN ^1^	GRASS ^1^	SEM ^2^	*p*-Value ^3^
Objective Color: lean tissue at ribeye area ^4^					
	L*	37.56	36.62	0.189	<0.0001
	a*	25.20	23.21	0.195	<0.0001
	b*	9.84	8.62	0.127	<0.0001
Objective Color: subcutaneous backfat ^5^					<0.0001
	L*	74.00	77.20	0.429	<0.0001
	a*	4.32	2.90	0.166	<0.0001
	b*	14.51	21.92	0.336	<0.0001
Ultimate pH ^6^		5.58	5.59	0.016	0.8051

^1^ Treatments: GRAIN = bison heifers (*n* = 108) backgrounded on grain and finished for 130 days with ad libitum access to grass hay, alfalfa, and a corn and dry distiller’s grain concentrate prior to slaughter. GRASS = bison heifers (*n* = 93) remained on pasture until slaughter. ^2^ Standard error of the mean. ^3^ Probability of difference among least square means. ^4^ Objective color measurement recorded on the exposed ribeye following an approximately 30 min bloom time; L*: 0 = Black, 100 = White; a*: Negative values = green; Positive values = red; b*: Negative values = blue; Positive values = yellow. ^5^ Objective color measurement of subcutaneous fat recorded on the external surface of the carcass, opposite the ribeye; L*: 0 = Black, 100 = White; a*: Negative values = green; Positive values = red; b*: Negative values = blue; Positive values = yellow. ^6^ Ultimate pH was measured on at either 2 or 3 days postmortem from grain- (*n* = 30) and grass- (*n* = 30) finished striploins.

**Table 3:** Least square means for the effect of finishing system on the proximate nutrient composition of raw tissue from the *longissimus dorsi* of grain- or grass-finished bison heifers.

Nutrient	GRAIN ^1^	GRASS ^1^	SEM ^2^	*p*-Value ^3^
Moisture, %	74.05	75.94	0.239	<0.0001
Protein, %	21.39	21.00	0.166	0.0221
Fat, %	3.21	1.94	0.227	<0.0001
Ash, %	1.08	1.09	0.010	0.2208
Cholesterol, (mg/100g)	54.31	51.41	1.043	0.0073

^1^ Treatments: GRAIN = bison heifers (*n* = 30) backgrounded on pasture and finished for 130 days with ad libitum access to grass hay, alfalfa, and a corn and dry distiller’s grain concentrate prior to slaughter. GRASS = bison heifers (*n* = 29) remained on pasture until slaughter. ^2^ Standard error of the mean. ^3^ Probability of difference among least square means.

**Table 4:** Least square means for the effect of finishing system on the fatty acid composition (mg/g wet sample basis) of raw tissue from bison *longissimus dorsi* of grain- or grass-finished bison heifers.

Fatty Acids	GRAIN ^1^	GRASS ^1^	SEM ^2^	*p*-Value ^3^
C10:0	0.02	0.01	0.003	0.0344
C12:0	0.02	0.02	0.002	0.2322
C14:0	0.49	0.31	0.033	<0.0001
C14:1n5	0.13	0.11	0.008	0.0057
C15:0	0.15	0.12	0.009	0.0013
C16:0	5.78	3.38	0.428	<0.0001
C16:1trans	0.11	0.11	0.010	0.8680
C17:0	0.38	0.23	0.032	<0.0001
C17:1	0.36	0.17	0.044	<0.0001
C18:0	3.85	2.71	0.285	0.0002
C20:0	0.09	0.26	0.012	<0.0001
C18:1n9cis	14.19	7.34	1.047	<0.0001
C18:1trans	0.25	0.21	0.019	0.0771
C18:1n7 *	-----	-----	-----	-----
C24:1n9	0.19	0.14	0.027	0.0512
C18:2trans	0.08	0.07	0.006	0.1741
C18:2n6	1.72	1.27	0.059	<0.0001
C18:3n3	0.25	0.27	0.017	0.1500
C18:3n6 *	-----	-----	-----	-----
C20:2	0.09	0.08	0.014	0.6545
C20:3n6	0.05	0.05	0.010	0.9112
C20:4n6	0.69	0.58	0.031	0.0009
C22:3	0.16	0.15	0.016	0.3935
C22:5n3	0.45	0.55	0.026	0.0008
C22:6n3	0.61	0.59	0.099	0.8703
TOTAL	30.97	19.07	1.984	<0.0001
SFA	10.80	7.03	0.780	<0.0001
MUFA	16.07	8.42	1.159	<0.0001
PUFA	4.11	3.62	0.196	0.0155
PUFA:SFA	0.41	0.58	0.046	0.0006
n-6:n-3 ratio	5.74	4.64	0.201	<0.0001

* Fatty acids present in minimal amounts that were undetected by gas chromatography analysis. ^1^ Treatments: GRAIN = bison heifers (*n* = 30) backgrounded on pasture and finished for 130 days with ad libitum access to grass hay, alfalfa, and a corn and dry distiller’s grain concentrate prior to slaughter. GRASS = bison heifers (*n* = 29) remained on pasture until slaughter. ^2^ Standard error of the mean. ^3^ Probability of difference among least square means.

**Table 5:** Least square means for the effect of finishing system on the fatty acid composition (%, g/100 g total fatty acids) of raw tissue from bison *longissimus dorsi* of grain- or grass-finished bison heifers.

Fatty Acids	GRAIN ^1^	GRASS ^1^	SEM ^2^	*p*-Value ^3^
C10:0	0.06	0.07	0.010	0.3869
C12:0	0.08	0.12	0.012	0.0020
C14:0	1.58	1.63	0.045	0.2631
C14:1n5	0.43	0.60	0.031	<0.0001
C15:0	0.49	0.64	0.030	<0.0001
C16:0	18.57	17.27	0.482	0.0092
C16:1trans	0.36	0.57	0.014	<0.0001
C17:0	1.21	1.17	0.042	0.3380
C17:1	1.12	0.85	0.116	0.0225
C18:0	12.35	14.11	0.347	<0.0001
C20:0	0.33	1.42	0.070	<0.0001
C18:1n9cis	45.60	37.38	0.925	<0.0001
C18:1trans	0.81	1.14	0.041	<0.0001
C18:1n7 *	-----	-----	-----	-----
C24:1n9	0.60	0.80	0.114	0.0791
C18:2trans	0.24	0.38	0.015	<0.0001
C18:2n6	5.94	7.24	0.457	0.0064
C18:3n3	0.86	1.55	0.117	<0.0001
C18:3n6 *	-----	-----	-----	-----
C20:2	0.26	0.47	0.026	<0.0001
C20:3n6	0.14	0.30	0.028	<0.0001
C20:4n6	2.32	3.33	0.220	<0.0001
C22:3	0.51	0.85	0.063	<0.0001
C22:5n3	1.58	3.10	0.192	<0.0001
C22:6n3	1.82	3.28	0.333	<0.0001
SFA	34.66	36.39	0.732	0.0219
MUFA	51.58	43.07	0.963	<0.0001
PUFA	13.75	20.53	1.219	<0.0001

* Fatty acids present in minimal amounts that were undetected by gas chromatography analysis. ^1^ Treatments: GRAIN = bison heifers (*n* = 30) backgrounded on pasture and finished for 130 days with ad libitum access to grass hay, alfalfa, and a corn and dry distiller’s grain concentrate prior to slaughter. GRASS = bison heifers (*n* = 29) remained on pasture until slaughter. ^2^ Standard error of the mean. ^3^ Probability of difference among least square means.

**Table 6:** Least squares means for effect of storage conditions on tenderness of striploin steaks from grain- and grass-finished bison.

Variable	FRESH ^1^	FROZEN ^1^	SEM ^2^	*p*-Value ^3^
WBSF, kg	3.24	2.72	0.526	<0.0001
Cook loss, %	20.71	22.67	0.356	<0.0001

^1^ Treatments: FRESH = striploin steaks (*n* = 60) from grain- and grass-finished bison heifers, aged 14 days, and kept in fresh storage conditions prior to analysis. FROZEN = striploin steaks (*n* = 60) from grain- and grass-finished heifers, aged 14 days and kept in frozen storage approximately 3 months prior to analysis. ^2^ Standard error of the mean. ^3^ Probability of difference among least square means.

**Table 7:** Least square means for the effect of finishing system on subjective meat quality attributes rated by a consumer sensory panel (*n*=113 participants).

Attribute ^1^	GRAIN ^2^	GRASS ^2^	SEM ^3^	*p*-Value ^4^
Overall liking	80.39	78.48	1.657	0.2591
Aroma liking	76.99	75.31	1.853	0.3756
Flavor liking	79.12	77.68	1.840	0.4426
Texture liking	79.88	77.23	2.212	0.2440
Toughness	6.64	7.32	0.519	0.2073
Juiciness	8.91	9.42	0.556	0.3693
Off-flavor	3.65	4.21	0.409	0.1861

^1^ Liking ratings were made on 0-120-point labeled affective magnitude scales, with the left most end (score of 0) labeled greatest imaginable disliking and the right most end (score of 120) labeled greatest imaginable liking. Intensity ratings were made on 0-20-point line scales with the left most ends labeled none (score of 0) and the right most ends labeled extremely intense for off flavor, extremely tough, or extremely juicy (score of 20). ^2^ Treatments: GRAIN = bison heifers backgrounded on pasture and finished for 130 days with ad libitum access to grass hay, alfalfa, and a corn and dry distiller’s grain concentrate prior to slaughter. GRASS = bison heifers remained on pasture until slaughter. ^3^ Standard error of the mean. ^4^ Probability of difference among least square means.

## Data Availability

The data presented in this study are available on request from the corresponding author. The data are not publicly available due to privacy restrictions.
